# Altered Neuronal Firing Pattern of the Basal Ganglia Nucleus Plays a Role in Levodopa-Induced Dyskinesia in Patients with Parkinson’s Disease

**DOI:** 10.3389/fnhum.2015.00630

**Published:** 2015-11-25

**Authors:** Xiaoyu Li, Ping Zhuang, Yongjie Li

**Affiliations:** ^1^Department of Neurosurgery, Xuanwu Hospital, Capital Medical UniversityBeijing, China; ^2^Beijing Institute of Functional Neurosurgery, Xuanwu Hospital, Capital Medical UniversityBeijing, China

**Keywords:** Parkinson’s disease, levodopa, movement disorders, globus pallidus, subthalamic nucleus, microelectrodes

## Abstract

**Background**: Levodopa therapy alleviates the symptoms of Parkinson’s disease (PD), but long-term treatment often leads to motor complications such as levodopa-induced dyskinesia (LID).

**Aim**: To explore the neuronal activity in the basal ganglia nuclei in patients with PD and LID.

**Methods**: Thirty patients with idiopathic PD (age, 55.1 ± 11.0 years; disease duration, 8.7 ± 5.6 years) were enrolled between August 2006 and August 2013 at the Xuanwu Hospital, Capital Medical University, China. Their Hoehn and Yahr (1967) scores ranged from 2–4 and their UPDRS III scores were 28.5 ± 5.2. Fifteen of them had severe LID (UPDRS IV scores of 6.7 ± 1.6). Microelectrode recording was performed in the globus pallidus internus (GPi) and subthalamic nucleus (STN) during pallidotomy (*n* = 12) or STN deep brain stimulation (DBS; bilateral, *n* = 12; unilateral, *n* = 6). The firing patterns and frequencies of various cell types were analyzed by assessing single cell interspike intervals (ISIs) and the corresponding coefficient of variation (CV).

**Results**: A total of 295 neurons were identified from the GPi (*n* = 12) and STN (*n* = 18). These included 26 (8.8%) highly grouped discharge, 30 (10.2%) low frequency firing, 78 (26.4%) rapid tonic discharge, 103 (34.9%) irregular activity, and 58 (19.7%) tremor-related activity. There were significant differences between the two groups (*p* < 0.05) for neurons with irregular firing, highly irregular cluster-like firing, and low-frequency firing.

**Conclusion**: Altered neuronal activity was observed in the basal ganglia nucleus of GPi and STN, and may play important roles in the pathophysiology of PD and LID.

## Introduction

Parkinson’s disease (PD) is characterized by progressive movement disorders including bradykinesia, resting tremor, rigidity, and loss of postural reflexes (Lees et al., 2009). The prevalence of PD increases with age (Pringsheim et al., 2014). In China, the prevalence of PD ranges from 16–440.3/1,00000, with an annual incidence ranging from 1.5–8.7/1,00000 (Zou et al., 2015). The etiology of PD is still poorly understood, but possible risk factors include pesticide exposure, head trauma, family history, drugs, hypertension, diabetes, hypercholesterolemia, hyperhomocysteinemia, carotid atherosclerotic plaques, and cancers (Chabolla et al., 1998; Dick et al., 2007; Gao et al., 2009; Li et al., 2015). Symptoms are caused by a loss of dopaminergic neurons in the substantia nigra.

Levodopa is considered the first-line treatment for PD (Pahwa et al., 2006). However, patients with advanced PD often react to levodopa with abnormal involuntary movements known as levodopa-induced dyskinesia (LID; Marsden, 1994). LID encompasses a variety of motor signs including choreic, ballistic, or dystonic movements (Fahn, 2000). These movements are important causes of disability in patients with PD (Marsden, 1994). The predominant activity of the basal ganglia during LID opposes the Parkinsonian state (Alexander et al., 1986; Albin et al., 1989; DeLong, 1990; Suarez et al., 2014).

Interestingly, administration of levodopa or apomorphine to monkeys with PD is associated with significantly reduced firing rate of globus pallidus internus (GPi) and subthalamic nucleus (STN) neurons during dyskinesia (Mitchell et al., 1992; Papa et al., 1999). Similar findings were observed in patients with PD receiving apomorphine (Lozano et al., 2000; Levy et al., 2001). Therefore, LID may result from decreased neuronal firing of GPi neurons, causing the loss of their normal inhibitory effects on thalamic-cortical motor projections, and the subsequent abnormal movements (Albin et al., 1989; DeLong, 1990; Vitek and Giroux, 2000).

Subsequent studies indicated that LID is not only associated with reduced firing rates but also with an abnormal firing pattern (Lozano et al., 2000; Levy et al., 2001). At the onset of apomorphine-induced dyskinesia, the firing of GPi neurons changes to an irregular pattern in patients with PD (Merello et al., 1999). Supportive data also come from the study of local field potentials using electrodes implanted in the GPi and STN after DBS surgery: abnormal patterns of oscillatory activity were observed off and on motor conditions (Foffani et al., 2005). These data suggest that changes in the pattern of oscillatory activity, rather than modifications in the firing rate alone, are involved in PD dyskinesia (Obeso et al., 2000), but decreased GPi firing rates cannot completely explain LID because lesions of GPi or stimulation both silence these neurons and lead to LID abolition (Obeso et al., 2000). Thus, changes in parameters of GPi and STN neuronal activity other than the firing rate might account for LID. Indeed, recordings obtained during GPi or STN exploration in patients with PD undergoing functional stereotactic surgery might provide a unique opportunity to examine the firing rates and patterns of neurons in the GPi or STN (Hutchison et al., 1994, 1998). Despite the impressive wealth of data reported by the above studies, the role of neuronal firing pattern of the basal ganglia nucleus in LID in PD patients remains unclear. Therefore, the present study aimed to assess neuronal activity in the basal ganglia nuclei in PD patients with LID. The neuronal activity in the GPi and STN from PD patients with or without severe LID was evaluated during the operation. We observed an altered neuronal activity in the basal ganglia nucleus of GPi and STN, indicating the important role of such changes in the pathophysiology of PD and LID. These findings broaden the current understanding of the pathophysiology of PD associated with LID.

## Materials and Methods

### Patients

Thirty patients with idiopathic PD (11 females; 19 males; 55.1 ± 11.0 years) undergoing surgery for PD were recruited. The diagnosis of PD was based on medical history, physiological and neurological examinations, and response to levodopa or dopaminergic drugs. All patients had tremor, rigidity, and bradykinesia. They were assessed using the Unified Parkinson’s Disease Rating Scale (UPDRS; Lang and Fahn, 1989) and the Hoehn and Yahr disability scale (Hoehn and Yahr, 1967) while being off medication. Surgery was performed 12 h after PD medication discontinuation. In addition, UPDRS IV (dyskinesia scale) scores were determined.

Inclusion criteria were: (1) primary PD; (2) regular treatment with levodopa; and (3) complete clinical records of therapeutic effect evaluation and intraoperative electrophysiological records. Exclusion criteria were: (1) secondary PD; (2) no levodopa treatment; or (3) incomplete clinical records of therapeutic effect evaluation and intraoperative electrophysiological records.

The diagnosis of LID is challenging because of the heterogeneity of its manifestations. Patients may show chorea or choreoathetosis, though myoclonus, akathasia, ballism, and other forms of involuntary movements. LID generally appears first on the side most affected by PD (Thanvi et al., 2007).

All clinical assessments were carried out by the same neurologist with >9 years of experience. Of the 30 patients with PD, 15 had severe LID, while LID was not detected in the remaining 15 individuals. The clinical characteristics of patients in both groups are shown in Table 1. A total of 12 patients (6 with LID and 6 without LID) underwent unilateral pallidotomy and 18 patients (9 with LID and 9 without LID) were subjected to STN deep brain stimulation (DBS; bilateral, *n* = 12; unilateral, *n* = 6; Medronic Inc., USA). Patients without LID were used as controls since the appearance of LID correlates with FosB expression in the denervated striatal areas (Darmopil et al., 2009; Suarez et al., 2014).

**Table 1:** **Characteristics of the patients with PD according to LID status**.

Characteristics	With LID	Without LID	*p*
Age at onset (years)	50.0 ± 9.8	61.1 ± 9.6	<0.05
Duration of disease (years)	10.9 ± 5.9	6.5 ± 4.5	<0.05
Duration of levodopa treatment (years)	9.1 ± 3.3	3.2 ± 2.0	<0.05
Dose of levodopa (mg)	960 ± 322	483 ± 215	<0.05
LID score	5.2 ± 3.1	0	<0.05

*PD, Parkinson’s disease; LID, levodopa-induced dyskinesia*.

The study was approved by the Ethics Committee of Xuanwu Hospital, Capital Medical University, China, and performed according to all Chinese and international regulations pertaining to human research, as stated in the Declaration of Helsinki. Written informed consent was obtained from each patient.

### Stereotactic Surgery and Neurophysiology

Surgeries were performed as previously described (Hutchison et al., 1994, 1998; Lang et al., 1997; Rodriguez-Oroz et al., 2001; Zhuang et al., 2004; Zheng et al., 2010). Medication was stopped 12 h before the operation, which was carried out by two surgeons with >10 years experience.

Briefly, after local anesthesia (lidocaine) at the pin sites, a stereotactic frame (CRW-FN, Radionics, Burlington, MA, USA) was affixed to the patient’s head in the supine position. A fiducial box and a magnetic resonance imaging (MRI) adapter were then attached to the frame. Sagittal MRI (Siemens 1.5 Tesla, Sonata, Germany) sequences for whole brain were obtained using rapid acquisition gradient echo. The Syngo LEONADO workstation (Syngo VE 26A, Siemens, Germany) was used for three dimensional reconstructions and target calculation based on the stereotactic atlas of Schaltenbrand and Wahren (Schaltenbrand and Wahren, 1977). The coordinates of anterior and posterior commissures (AC and PC, respectively) in relation to the center of the stereotactic frame were identified and adjusted, making them coincide with the length of the intercommissural line of the patient. In the present study, the coordinates of the target GPi were 2 mm anterior to the midpoint of the ACPC line, 4–6 mm below the ACPC line, and 18–22 mm lateral to the midline. The coordinates of the target STN were 1–2 mm posterior to the midpoint of the ACPC line, 4–6 mm below the ACPC line, and 9–12 mm lateral to the midline.

Intraoperative electrophysiological recordings were made using microprobes, except for the STN, in which deep electrodes were used. Physiological confirmation of the GPi was achieved using microelectrode recordings. The presence of “border cells” (Hutchison et al., 1994; Lang et al., 1997; Zhuang et al., 2004) marking the boundaries of the motor nerve nuclear segment, the characteristic discharge patterns of neurons in globus pallidus externa (GPe) and GPi (including GPie and GPii) and in the surrounding regions (GPe, lateral part of GPi, and medial part of GPi), and location of cell-dense vs. cell-sparse zones allowed the discrimination of the various pallidal segments. The final target landmark was the location of the optic tract (OT) border, as confirmed using strobe light and microelectrode stimulation. For the STN, neuronal activity of a typical recording track corresponded initially to the striatum (caudate nucleus) followed by low amplitude fiber activity while passing through the internal capsule.

Thalamic activity, corresponding to the reticular nucleus, was recorded when an angle of 60° was used and when the track was relatively caudal (Rodriguez-Oroz et al., 2001). Upon entering the STN, a robust increase in neuronal activity was encountered, with multiple units discharging at relatively high frequency. Such activity may be recorded for a length between 1 and 7 mm, depending on the relative position of the recording electrode in the mediolateral and rostrocaudal planes of STN. The bottom of the nucleus was indicated by a reduced number of active neurons followed by electrical silence for 0.5–3 mm depending on the laterality. This was followed by tonic, high frequency neuronal activity, which corresponds to the substantia nigra pars reticulata (SNr; Hutchison et al., 1998; Rodriguez-Oroz et al., 2001; Zhuang et al., 2004).

Microelectrode recording started at a distance of 10 mm from the final target in the anterosuperior position. Extracellular action potential signals were amplified (×20,000), filtered (with a bandpass of 100–5 kHz), and fed to an audio monitor with an AC amplifier (FHC, Inc., Bowdoinham, ME, USA).

Simultaneously with a channel of microelectrode recording, three channels of EMG were recorded using surface electrodes on contralateral muscle groups relevant to the tics including extensor carpi radialis (ECR), flexor carpi radialis (FCR), and tibialis anterior (TA) using PolyView 2.5 (Astro-Med. Inc., RI, USA). The signal was sampled at 7.5 kHz and displayed on a computer screen and an oscilloscope (HITACHI, V-1560, Japan). Only well-isolated and stable single units recorded between 15 s to several minutes were analyzed. Data were stored for off-line analysis.

Throughout the surgical procedure, all patients were required to be awake and conscious to cooperate with the neurosurgeon. They were assessed using physical monitoring (e.g., speech was assessed by verbal task such as sentence repetition and counting numbers; tone was monitored by passively moving the limbs, performing a simple movement, or holding a certain posture) to avoid complications and to evaluate the effect of target lesion on limbs. Pallidotomy and STN DBS were carried out as previously described in Benabid et al. (1994) and Lozano et al. (1995).

### Data Analysis

For neuronal data analysis, only spikes (negative upward) with signal-to-noise ratios >2 were used. Action potentials were confirmed to arise from a single cell by amplitude and shape criteria. The confirmation method included examining whether the shape of the action potential was constant, as verified by displaying the shape on the screen at a time window of at least 15–20 s (Lang and Fahn, 1989; Rodriguez-Oroz et al., 2001). The interspike intervals (ISIs) were manually measured and ISI histograms were constructed to evaluate the pattern of neuronal discharges. Mean firing rates and corresponding standard deviation (SD) values were determined. The degree of regularity of neuronal discharge was determined by calculating the coefficients of variation (CVs) of ISIs as CV = SD of ISIs/mean ISIs (Rodriguez-Oroz et al., 2001). PolyView 2.5 (Astro-Med. Inc., USA) and Origin 7.0 (OriginLab Corporation, USA) were used for the analyses, which were performed in a blinded fashion.

### Clinical Outcome Evaluation

Motor and dyskinesia scores (UPDRS III and IV, Chinese versions) were used to evaluate PD symptoms during the off medication state, as well as dyskinesia symptoms pre- and post-surgery, and at 6 months. All assessments were made by the same evaluator.

### Statistical Analysis

Normally distributed continuous variables are presented as mean ± SD and were analyzed using one-way analysis of variance (ANOVA) with Bonferroni correction and Least Significant Difference (LSD) *post hoc* tests. Non-normally distributed continuous variables are presented as median (interquartile range) and were analyzed using the Mann-Whitney U test. Categorical data are presented as frequencies and were analyzed using the chi-square or the Fisher’s exact test, as appropriate. SPSS 12.0 (SPSS Inc., Chicago, IL, USA) and Origin 7.0 (OriginLab Corporation, USA) were used for statistical analyses. Two-sided *p*-values < 0.05 were considered statistically significant.

## Results

### Neurons Assessed

The number of assessed neurons differed from one patient to another, and was random and not determined by surgeons; as many neurons as possible were assessed during surgery. Therefore, 295 neurons were obtained from recording trajectories of GPi (*n* = 12) and STN (*n* = 18). The difference in the number of patients in the two groups is because STN recordings were obtained by either two-channel or single-channel of electrode track, while GPi was recorded using a single electrode only.

### Neuronal Activity in the GPi

**Figure 1:**
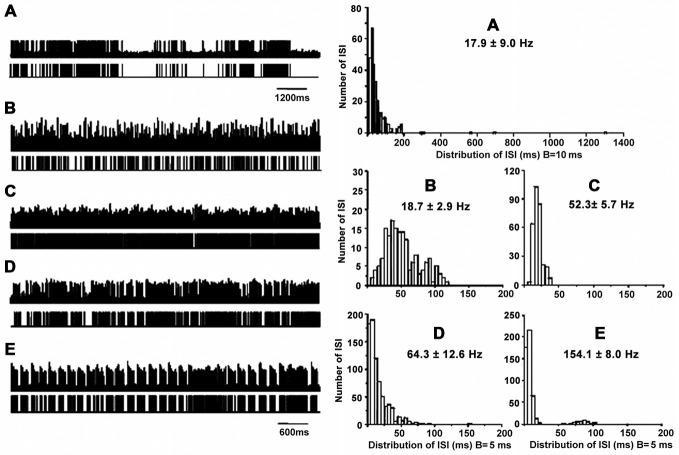
**Patterns of neuronal discharge with corresponding spike trains and ISI histograms representative for GPi neurons in patients with PD and LID.** Five patterns were obtained from the GPi (*n* = 12 patients), including neurons with grouped discharge **(A)**, low frequency firing **(B)**, tonic neuronal firing **(C)**, irregular neuronal firing **(D)**, and tremor-related neuronal activity **(E)**. Discharge frequencies were 17.8 ± 9.0, 18.7 ± 2.9, 52.3 ± 5.7, 64.3 ± 12.6 and 154.1 ± 8.0 Hz, respectively, for types A–E neurons.

A total of 80 neurons were obtained from the GPi (*n* = 12), of which 7 (8.8%) showed grouped discharge following long pauses, 12 (15.0%) showed a low firing rate, 20 (25.0%) showed rapid tonic firing, 22 (27.5%) showed irregular neuronal activity, and 19 (23.8%) showed tremor-related neuronal activity (*p* < 0.01). Figure 1 illustrates the five patterns of neuronal discharge with the corresponding spike trains and ISI histograms representative for GPi neurons in parkinsonian patients with or without LID.

Cells A showed highly irregular activity with grouped discharge separated by pauses, unlike the pattern of cells D, which had irregular discharge in PD. As for cells B, lower tonically firing frequency was observed compared to cells C, which are neurons with rapid tonically firing often seen in PD. The pattern of cells E was tremor-related, as often found in PD. The ISI histograms confirmed that cells A had a broader range of ISI time (1200 ms) compared to the other cells. In particular, cells A and B had lower mean firing rates (18–19 Hz) compared to cells C and D (50–150 Hz; Figure 1). The data were collected from patients with LID, and discharge frequencies were 17.9 ± 9.0 Hz (cells A), 18.7 ± 2.9 Hz (cells B), 52.3 ± 5.7 Hz (cells C), 64.3 ± 12.6 Hz (cells D), and 154.1 ± 8.0 Hz (cells E).

Further analysis revealed ISI values were 35 ± 50 ms with CVs of 1.6 ± 0.5 and firing rates of 31 ± 12 Hz for neurons with grouped discharge, ISI values of 52 ± 51 ms were obtained for cells with low firing frequency, with CVs of 0.7 ± 0.2 and firing rates of 18 ± 6 Hz, For tonic firing cells, ISI values were 19 ± 8 ms, with CVs of 0.4 ± 0.1 and firing rates of 60 ± 38 Hz, ISI values of 19 ± 20 ms were obtained for neurons with irregular activity; the corresponding CVs were 0.8 ± 0.3 with firing rates of 63 ± 15 Hz. Finally, ISI values of 11 ± 14 ms with CV of 1.2 ± 0.4, and firing rates of 82 ± 42 Hz for neurons with tremor-related neuronal activity. There were statistically significant differences in ISI values and CVs between the five cell groups (*p* < 0.05; Table 2).

**Table 2:** **Different patterns of neuronal activity in the GPi**.

Pattern of neuronal activity	Number of cells	Frequency (Hz)	ISI (ms)	CV of ISI
Grouped discharge	7	31 ± 12 (18–51)	35 ± 50 (2–1309)	1.6 ± 0.5 (1.0–2.1)
Low frequency	12	18 ± 6 (9–29)	52 ± 51 (25–366)	0.7 ± 0.2 (0.2–1.4)
Tonic	20	60 ± 38 (37–176)	19 ± 8 (3–79)^△^	0.4 ± 0.1 (0.4–0.5)^△^
Irregular	22	63 ± 15 (40–93)	19 ± 20 (11–229)^△^	0.8 ± 0.3 (0.7–1.2)^△^
Tremor-related	19	82 ± 42 (30–135)	11 ± 14 (12–117)	1.2 ± 0.4 (0.7–1.7)

*^△^ Indicates significance (Bonferroni test, p < 0.05). Neurons with tonic firing were compared with neurons with low frequency firing. Neurons with irregular discharge were compared with neurons with grouped discharge. Values in brackets represent ranges. GPi, globus pallidus internus; ISI, interspike interval; CV, coefficient of variation*.

### Neuronal Activity in the STN

**Figure 2:**
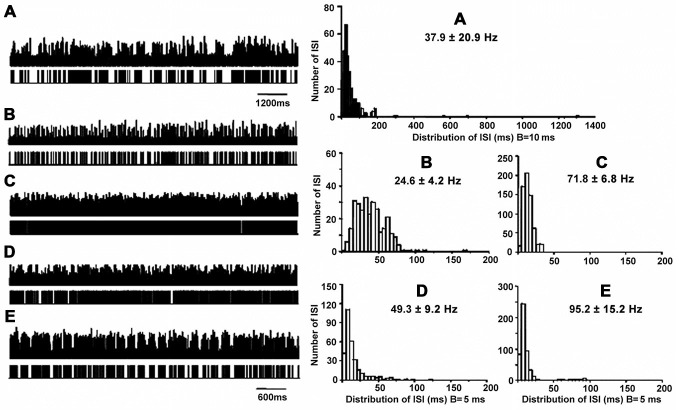
**Patterns of neuronal discharge with corresponding spike trains and ISI histograms representative for STN neurons in patients with PD and LID.** Five patterns were obtained from STN (*n* = 18 patients), including neurons with grouped discharge **(A)**, low frequency firing **(B)**, tonic neuronal firing **(C)**, irregular neuronal firing **(D)**, and tremor-related neuronal activity **(E)**. Discharge frequencies were 37.9 ± 20.9, 24.6 ± 4.2, 71.8 ± 6.8, 49.3 ± 9.2 and 95.2 ± 15.2 Hz, respectively, for types A–E neurons.

A total of 215 neurons were obtained from STN (*n* = 18), of which 19 (8.8%) showed grouped discharge following long pauses, 18 (8.4%) showed low firing rate, 58 (27.0%) showed rapid tonic firing, 81 (37.7%) showed irregular neuronal activity, and 39 (18.1%) showed tremor-related neuronal activity (*p* < 0.01). Figure 2 illustrates the five patterns of neuronal discharge with corresponding spike trains and ISI histograms representative for STN neurons in patients with PD and LID. Similar to Figure 1, patterns of A–B were found in patients with PD and LID, while patterns of C–E were usually obtained in PD. The different patterns have been described above for the GPi. However, types A and B cells had firing rates of 25–38 Hz, while the values obtained for C–D were 50–95 Hz (Figure 2). The data were collected from patients with LID, and discharge frequencies were 37.9 ± 20.9 Hz (cells A), 24.6 ± 4.2 Hz (cells B), 71.8 ± 6.8 Hz (cells C), 49.3 ± 9.2 Hz (cells D) and 95.2 ± 15.2 Hz (cells E).

Further ISI analysis revealed that ISI values were 35 ± 71 ms with CVs of 2.0 ± 0.5 and firing rates of 28 ± 8 Hz for neurons with grouped discharge. As for cells with low firing frequency, ISI values of 52 ± 46 ms were obtained with CVs of 0.7 ± 0.2; firing rates were 18 ± 6 Hz, ISI values of 14 ± 7 ms with CVs of 0.4 ± 0.2 were obtained for tonic firing cells, which had firing rates of 71 ± 16 Hz. For neurons with irregular activity, ISI values were 25 ± 20 (*CV* = 0.8 ± 0.3), and they had firing rates of 45 ± 16 Hz. ISI values were 12 ± 16 ms with CVs of 1.1 ± 0.3, and firing rates of 74 ± 29 Hz for neurons with tremor-related neuronal activity. These results were similar to those obtained in GPi, also indicating statistically significant differences in ISI values and CVs among the five cell groups (*p* < 0.01; Table 3).

**Table 3:** **Different patterns of neuronal activity in STN**.

Pattern of activity	Numbers of cells	Frequency (Hz)	ISI (ms)	CV of ISI
Grouped discharge	19	28 ± 8 (12.9–37.9)	35 ± 71 (22–1254)	2.0 ± 0.5 (1.3–3.0)
Low frequency	18	18 ± 6 (8.0–23.3)	52 ± 46 (5–310)	0.7 ± 0.2 (0.3–1.4)
Tonic	58	71 ± 16 (56–90)	14 ± 7 (3–49)^#^	0.4 ± 0.2 (0.2–0.5)^#^
Irregular	81	45 ± 16 (19.3–68.5)	25 ± 20 (15–278)^#^	0.8 ± 0.3 (0.4–1.5)^#^
Tremor-related	39	74 ± 29 (53–114)	12 ± 16 (31–152)	1.1 ± 0.3 (0.4–1.4)

*^#^Indicates significance (Bonferroni test, p < 0.05), Neurons with tonic firing compared neurons with low frequency firing. Neurons with irregular discharge compared with neurons with grouped discharge. Values in brackets represent ranges. STN, subthalamic nucleus; ISI, interspike interval; CV, coefficient of variation*.

### Neurons with Grouped Discharge and Low Frequency Firing in GPi and STN of Patients with and Without LID

The majority of neurons with grouped discharge and those with low firing frequency were found in patients with LID. There were significant differences when comparing neurons with grouped discharge and those with low firing rates between the patients with LID (GPi and STN) and subjects without LID (GPi; *p* < 0.01; Table 4).

**Table 4:** **Numbers of neurons with grouped discharge and low firing rates in GPi and STN in patients with LID and without LID**.

Type of cell	PD with LID (GPi: *n* = 6)	PD without LID (GPi: *n* = 6)	PD with LID (STN: *n* = 15)	PD without LID (STN: *n* = 15)
Grouped discharge	7 (16.3%)	0** (0%)	17 (16.0%)	2** (1.8%)
Low frequency firing	10 (23.3%)	2** (5.4%)	16 (15.1%)	2** (1.8%)
Total	43	37	106	109

**Indicates significance, (chi-square test P < 0.05) compared with patients without LID. GPi, globus pallidus internus; STN, subthalamic nucleus; ISI, interspike interval; CV, coefficient of variation; PD, Parkinson’s disease; LID, levodopa-induced dyskinesia.

### Clinical Outcome

**Figure 3:**
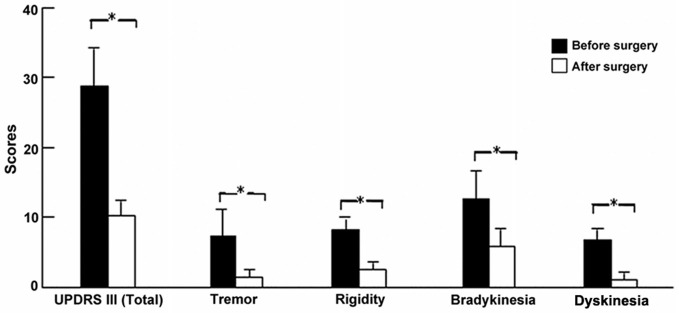
**Clinical outcomes of PD and LID symptoms after surgery.** *p < 0.01 (paired *t*-test) compared with before surgery in patients with LID.

UPDRS showed that the clinical improvements during the off-medication state for tremor, rigidity, and bradykinesia were 75.8, 73.6, and 52.5%, respectively in all patients (all *p* < 0.01, paired *t*-test). Similarly, the improvement of dyskinesia in patients with LID (*n* = 15) was 80.9% (*p* < 0.01; Figure 3).

## Discussion

Levodopa therapy can alleviate the symptoms of PD, but long-term treatment often leads to motor complications such as LID. The present study aimed to explore neuronal activity in the basal ganglia nuclei in patients with PD and LID. We identified 295 neurons from the GPi (*n* = 12) and STN (*n* = 18), of which 26 (8.8%) showed grouped discharge following long pauses, 30 (10.2%) showed low firing rate, 78 (26.4%) showed rapid tonic firing, 103 (34.9%) showed irregular neuronal activity, and 58 (19.7%) showed tremor-related neuronal activity. There were significant differences between the two groups for neurons with irregular firing, highly irregular cluster-like firing, and low-frequency firing. The altered neuronal activity observed in the basal ganglia nucleus of GPi and STN may play important roles in the pathophysiology of PD and LID.

In this study, all patients had idiopathic PD, of which 15 with severe LID constituted the study group; the remaining 15 individuals did not show any sign of LID and were used as controls (Darmopil et al., 2009; Suarez et al., 2014). Interestingly, significant differences of age at disease onset, disease duration, and levodopa dosage were observed between the two groups. Specifically, patients with LID were much younger than those without. These data corroborate recent findings that age at PD onset is the single most important risk factor for LID (Fabbrini et al., 2007).

According to the pathophysiological model of PD, dopamine depletion in PD leads to a series of functional changes that mediate the motor features of PD (Albin et al., 1989; DeLong, 1990; Vitek and Giroux, 2000). The most important characteristic is increased neuronal activity in the STN and GPi, resulting in excessive inhibition of the thalamocortical and brainstem motor nuclei; this leads to hypokinetic symptoms of PD such as bradykinesia, rigidity, and resting tremor, combined with on-time dyskinesia (Albin et al., 1989; DeLong, 1990; Hutchison et al., 1994, 1998; Rodriguez-Oroz et al., 2001; Reichmann, 2015). On the other hand, LID in PD and chorea-ballism as hyperkinetic symptoms are associated with reduced neuronal activity in basal ganglia output (Albin et al., 1989; DeLong, 1990; Vitek and Giroux, 2000). Neuronal activity in the GPi and STN shifts from increased firing in the parkinsonian state to marked hypoactivity during levodopa or apomophine-induced dyskinesia in MPTP monkeys (Mitchell et al., 1992; Papa et al., 1999) and patients with PD and LID (Lozano et al., 2000; Levy et al., 2001; Lee et al., 2007). In the present study, three neuronal activity types often found in PD were observed, in addition to low neuronal firing and high group discharge in GPi and STN as observed in LID studies. Although these results are preliminary and obtained during the off-medication state, they provide evidence that two opposite neuronal firing patterns coexisting in GPi and STN might be responsible for LID in patients with PD.

In this study, LID severity and PD symptoms were significantly improved after pallidotomy and STN DBS, in agreement with other clinical studies revealing that lesion of GPi and STN DBS induces motor improvement in PD, with a notable effect against LID (Lang et al., 1997; Guridi et al., 2008). These results confirm that interference at the level of GPi and STN can result in clinical improvement of PD and LID symptoms, supporting the view that basal ganglia dysfunction is involved in PD and LID (Alexander et al., 1986; Albin et al., 1989; DeLong, 1990; Vitek and Giroux, 2000).

The most direct and reliable data related to the functional state of dyskinesia come from electrophysiological studies. Filion and Tremblay (1991) assessed the effects of dopaminergic drugs on neuronal firing activity in the GPi of MPTP-treated monkeys. They found that apomorphine-induced dyskinesia is associated with a reduction in GPi firing rates. Subsequently, Papa et al. (1999) recorded the time course of LID induction in the GPi of monkeys with PD, and found that the firing rates of most neurons decreased from 46.3 Hz in the PD state to 7.6 Hz after LID occurrence. Hamada and Delong (1988) demonstrated that lesions in the STN of MPTP monkeys reduced the neuron activity in the GPi, which was associated with the appearance of dyskinesia. Boraud et al. (1998) reported that involuntary movements are induced by injection of dopamine receptor agonist together with changes of firing frequency and pattern in the GPi and STN in MPTP monkeys. A recent work demonstrated that the firing rate of striatal projection neurons increases in dyskinetic mice after D1R sensitization (Suarez et al., 2014), while multiple animal studies have shown that LID is related to D1 receptor super-sensitization (Pavon et al., 2006; Darmopil et al., 2009; Ruiz-Dediego et al., 2015; Solis et al., 2015), indicating the complexity of LID electrophysiology.

The present study is not without limitations. Indeed, the sample size was small and the study was carried out in a single center. In addition, only one method was used to assess neuronal functions, and the use of additional methods could provide more details regarding LID. Therefore, additional studies are necessary to correctly assess the electrophysiology of LID in PD.

In conclusion, the altered neuronal activity observed in the basal ganglia nucleus of the GPi and STN may play important roles in the pathophysiology of LID in PD. These findings provide additional understanding of LID, and could help better prevent this condition and improve the quality of life of PD patients with LID.

## Author Contributions

XYL participated in study design, data collection and analysis, and wrote the manuscript. PZ conceived of the study, participated in data collection and analysis, and provided critical revision. YJL participated in data and analysis, and provided critical revision. All authors read and approved the final manuscript, and agree to be accountable for all aspects of the work in ensuring that questions related to the accuracy or integrity of any part of the work are appropriately investigated and resolved.

## Funding

This work was supported by the National Scientific Foundation of China (No. 81171061, 81371256, 81361128012) and the Ministry of Education of the Republic of China (BIBD-PXM2013-014226-07-000084).

## Conflict of Interest Statement

The authors declare that the research was conducted in the absence of any commercial or financial relationships that could be construed as a potential conflict of interest.
